# The TGFβ-induced phosphorylation and activation of p38 mitogen-activated protein kinase is mediated by MAP3K4 and MAP3K10 but not TAK1

**DOI:** 10.1098/rsob.130067

**Published:** 2013-06

**Authors:** Gopal P. Sapkota

**Affiliations:** MRC Protein Phosphorylation and Ubiquitylation Unit, College of Life Sciences, University of Dundee, Dow Street, Dundee DD1 5EH, UK

**Keywords:** TAK1, transforming growth factor, SMAD, MAP3K10, MAP3K4, MLK2

## Abstract

The signalling pathways downstream of the transforming growth factor beta (TGFβ) family of cytokines play critical roles in all aspects of cellular homeostasis. The phosphorylation and activation of p38 mitogen-activated protein kinase (MAPK) has been implicated in TGFβ-induced epithelial-to-mesenchymal transition and apoptosis. The precise molecular mechanisms by which TGFβ cytokines induce the phosphorylation and activation of p38 MAPK are unclear. In this study, I demonstrate that TGFβ-activated kinase 1 (TAK1/MAP3K7) does not play a role in the TGFβ-induced phosphorylation and activation of p38 MAPK in MEFs and HaCaT keratinocytes. Instead, *RNAi*-mediated depletion of MAP3K4 and MAP3K10 results in the inhibition of the TGFβ-induced p38 MAPK phosphorylation. Furthermore, the depletion of MAP3K10 from cells homozygously knocked-in with a catalytically inactive mutant of MAP3K4 completely abolishes the TGFβ-induced phosphorylation of p38 MAPK, implying that among MAP3Ks, MAP3K4 and MAP3K10 are sufficient for mediating the TGFβ-induced activation of p38 MAPK.

## 2. Introduction

Members of the transforming growth factor beta (TGFβ) family of cytokines regulate a plethora of cellular processes, including growth control, differentiation, extracellular matrix production, migration, survival and apoptosis [1]. These pleotropic effects are due, in part, to the ability of the TGFβ cytokines to exert direct control over multiple signalling networks in addition to the control of the canonical SMAD-dependent signalling pathway [2,3]. Aberrations of both canonical and non-canonical signalling pathways downstream of the TGFβ cytokines often result in the manifestation of various human diseases, including fibrosis, cancer progression and metastasis [4,5].

TGFβ ligands initiate cellular responses by binding to their cognate type II and type I transmembrane receptor serine threonine protein kinases. Upon ligand binding, the type II receptor phosphorylates and activates the type I receptor kinase [1]. In the canonical pathway, the type I receptors then phosphorylate the SMAD transcription factors—1,5 and 8 in the bone morphogenetic protein (BMP) pathway, and 2 and 3 in the TGFβ pathway—which leads to their association with SMAD4 and entry into the nucleus. In the nucleus, together with the other transcription cofactors, SMADs control the expression of hundreds of target genes [1]. Some of the SMAD-independent pathways, often referred to as non-canonical pathways, that are directly modulated by the TGFβ ligands include various layers of the mitogen-activated protein kinase (MAPK) pathways, the PI3K/AKT/mTOR pathways and the RhoA-dependent signalling pathways [2]. A combination of the canonical and non-canonical signalling outputs, as well as context-dependent crosstalk inputs from other signalling networks, probably define the nature of cellular responses to TGFβ ligands [2,6–9].

Among the MAPK pathways activated by the TGFβ ligands, the activation of p38 MAPK has been the best characterized and widely investigated. Both SMAD-dependent and -independent mechanisms have been proposed for the TGFβ-induced phosphorylation and activation of p38 MAPK [10–17]. Inhibitors of p38 MAPK inhibit the TGFβ-induced epithelial-to-mesenchymal transition (EMT), as well as cell death, implying that the activation of p38 MAPK is crucial in regulating these cellular responses to TGFβ [14,16]. There are four mammalian p38 MAPK isoforms, namely p38α, p38β, p38δ and p38γ [18]. While unique roles for different isoforms have been reported [18,19], primarily most stimuli result in the activation of p38α MAPK by the MAP kinase kinases (MKKs) MKK3, MKK6 or MKK4 [20,21]. The MKKs phosphorylate the activation loop residues, Thr180 and Tyr182, of p38α MAPK [20]. Further upstream, MKKs are activated by various MAP3Ks in response to different stimuli [18,20]. The search for the MAP3Ks responsible for mediating the TGFβ-induced phosphorylation of p38 MAPK has generated great interest in the field and has led to numerous publications [11,15,17,22–24]. As the name indicates, TGFβ-activated kinase 1 (TAK1, also known as MAP3K7) was the first MAP3K reported to mediate the activation of p38 MAPK in response to TGFβ [17]. Subsequent reports have claimed that TGFβ receptor complexes bind to and activate TNF-receptor-associated factor 6 (TRAF6), resulting in its autoubiquitylation through K63-linked ubiquitin chains, and this allows TRAF6 to recruit and activate TAK1 [11,24]. Catalytically active TRAF6 is indispensable for mediating the interleukin-1 receptor (IL-1R) and toll-like receptor (TLR)-mediated activation of TAK1, and subsequent downstream signalling events such as the activation of p38 MAPK and the production of nuclear factor κB (NF-κB) and IFN regulatory factors [25,26]. However, autoubiquitylation of TRAF6 is unlikely to play a role in recruiting TAK1, as lysine-free TRAF6 has been demonstrated to restore IL-1-stimulated TAK1 activation to TRAF6^−/−^ cells [25]. Another MAP3K, MEKK4 (MAP3K4), has also been proposed to mediate the TGFβ-induced phosphorylation of p38 MAPK through SMAD-dependent expression of GADD45β, which associates with and activates MAP3K4 [15]. MEKK1 (MAP3K1) has been proposed to mediate the TGFβ-induced activation of c-Jun N-terminal kinase (JNK) isoforms [27].

In this paper, I examine the roles of various MAP3Ks in mediating the TGFβ-induced phosphorylation of p38 MAPK. I demonstrate that the loss of TAK1 (MAP3K7) in mouse embryonic fibroblasts (MEFs) or human keratinocytes (HaCaT) does not affect the levels of TGFβ-induced phosphorylation of p38 MAPK. Furthermore, restoring wild-type (WT) or catalytically inactive TAK1 in TAK1-deficient MEFs does not alter the ability of TGFβ to induce the phosphorylation of p38 MAPK. By using a comprehensive *RNAi* screen to knockdown all human MAP3Ks, I demonstrate that the depletion of MEKK4 (MAP3K4) and MLK2 (MAP3K10) results in a moderate reduction in the TGFβ-induced phosphorylation of p38 MAPK. The depletion of MLK2 (MAP3K10) in cells with homozygous knockin of catalytically inactive MEKK4 (MAP3K4) results in a complete loss of the TGFβ-induced phosphorylation of p38 MAPK, implying that MEKK4 and MLK2 mediate the TGFβ-induced phosphorylation and activation of p38 MAPK in MEFs and HaCaT keratinocytes.

## 3. Results

### 3.1. TAK1 (MAP3K7) does not mediate the TGFβ-induced phosphorylation of p38 MAPK

**Figure 1.**
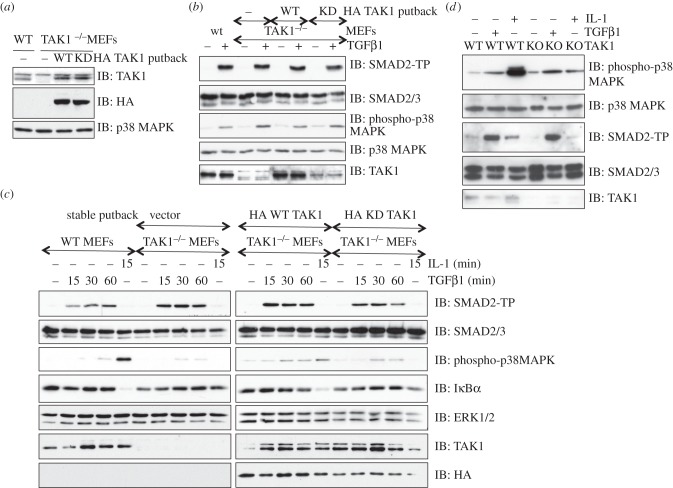
TGFβ induces phosphorylation of p38 MAPK in TAK1/MAP3K7-null MEFs. (*a*) Extracts (20 μg) from wild-type (WT) MEFs or TAK1-deficient (TAK1^−/−^) MEFs stably reintroduced with a control vector (−) or vectors encoding HA-tagged TAK1 (WT) or a catalytically inactive TAK1 (D175A) mutant (KD) were probed for protein expression with anti-TAK1, anti-HA and anti-p38 MAPK antibodies. (*b*) The cells described in (*a*) were starved for 16 h and treated with or without TGFβ (50 pM) for 45 min prior to lysis. Extracts were subjected to SDS–PAGE and immunoblotting with the indicated antibodies. (*c*) As in (*b*) except that a time course of TGFβ treatment was performed prior to lysis. Mouse IL-1α (5 ng ml^−1^, 10 min) treatment was used as a positive control to activate TAK1 in cells. Extracts (20 μg) were resolved by SDS–PAGE and analysed by immunoblotting using the indicated antibodies. WT and TAK1-deficient MEFs used in (*a–c*) above were provided by S. Akira, Osaka University, Japan. (*d*) Extracts (20 μg) from wild-type (WT) and TAK1-deficient (KO) MEFs (provided by S. Ghosh, Columbia University, New York, NY) treated with or without TGFβ (50 pM; 45 min) or mouse IL-1α (5 ng ml^−1^, 10 min) were resolved by SDS–PAGE and analysed by immunoblotting using the indicated antibodies.

In order to investigate the contribution of TAK1 in mediating the TGFβ-induced phosphorylation of p38 MAPK, I obtained WT and TAK1-deficient MEFs [28]. Additionally, using these cells, I generated TAK1-deficient MEFs stably expressing a control vector, or N-terminal HA-tagged human WT TAK1 or catalytically inactive (kinase dead, KD) TAK1 (figure 1*a*). Treatment of these cells with TGFβ for 45 min resulted in phosphorylation of SMAD2 to the same extent (figure 1*b*). Rather surprisingly, the levels of TGFβ-induced phosphorylation of p38 MAPK observed in TAK1^−/−^ MEFs were similar to those seen in WT MEFs (figure 1*b*). Restoration of WT TAK1 or KD TAK1 in TAK1-deficient MEFs did not result in significant changes to the levels of TGFβ-induced phosphorylation of p38 MAPK, albeit the expression of HA KD TAK1 was less than HA WT TAK1 (figure 1*b*). In order to determine whether TAK1 plays a role in modulating a time-dependent activation of p38 MAPK in response to TGFβ, a time course of TGFβ treatment was performed in these cells (figure 1*c*). No significant differences in TGFβ-induced phosphorylation of SMAD2 or p38 MAPK were observed in WT or TAK1-deficient MEFs, nor in TAK1-deficient MEFs restored with WT or KD TAK1 at any of the time points assayed (figure 1*c*). As expected, interleukin-1α (IL-1α) induced a robust p38 MAPK phosphorylation and loss of IκBα only in WT MEFs but not in TAK1-deficient MEFs (figure 1*c*). The IL-1α-induced p38 MAPK phosphorylation, and loss of IκBα was partially rescued in TAK1-deficient MEFs expressing HA WT TAK1 but not HA KD TAK1 (figure 1*c*).

In order to complement the above findings and definitively establish that TAK1 does not mediate the TGFβ-induced phosphorylation of p38 MAPK, I obtained a different set of MEFs from WT and TAK1-deficient mice that were generated independently using different targeting strategy [29]. By using these MEFs, I was able to demonstrate that there was no difference in the levels of TGFβ-induced p38 MAPK phosphorylation between WT and TAK1-knockout MEFs (figure 1*d*). TGFβ induced similar levels of SMAD2 phosphorylation in WT or TAK1-knockout MEFs (figure 1*d*). As expected, IL-1α-induced p38 MAPK was significantly inhibited in TAK1-knockout MEFs compared with the WT (figure 1*d*).

### 3.2. TGFβ does not activate TAK1

**Figure 2.**
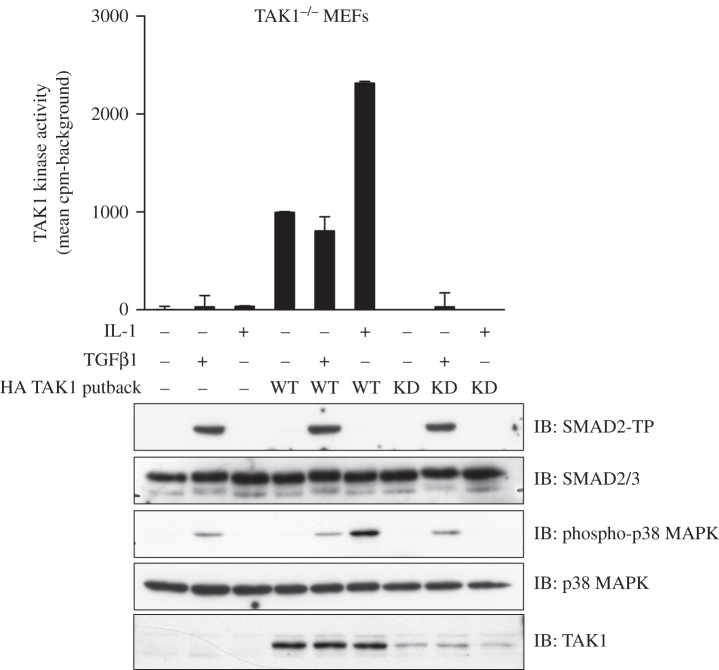
TGFβ does not activate TAK1: TAK1-deficient (TAK1^−/−^) MEFs stably reintroduced with a control vector (−) or vectors encoding HA-tagged TAK1 (WT) or a catalytically inactive TAK1 (D175A) mutant (KD) were treated with/without TGFβ (50 pM, 45 min) or mouse IL-1α (5 ng ml^−1^, 10 min) prior to lysis. Extracts (20 μg) were resolved by SDS–PAGE and immunoblotted with the indicated antibodies. *In vitro* TAK1 kinase assays were performed as described in §5.

Next, in order to assess whether TGFβ induces TAK1 kinase activity, I used an *in vitro* kinase assay developed for the measurement of TAK1 activity from cell extracts [30]. As expected, TGFβ or IL-1α did not stimulate any TAK1 activity in TAK1-deficient cells or TAK1-deficient cells stably expressing KD TAK1 (figure 2). In TAK1-deficient cells stably expressing WT TAK1, a basal TAK1 kinase activity was detected under ambient conditions (figure 2). Treatment of these cells with IL-1α stimulated a significant increase in TAK1 kinase activity (figure 2). However, treatment of these cells with TGFβ did not induce TAK1 activity over basal untreated conditions (figure 2). In all cases, TGFβ induced similar levels of p38 MAPK and SMAD2 phosphorylation. Treatment of cells with IL-1α resulted in the phosphorylation of p38 MAPK only in TAK1-deficient cells stably expressing WT TAK1 (figure 2), but not in TAK1-deficient cells or TAK1-deficient cells expressing KD TAK1 (figure 2).

### 3.3. TAK1 does not affect BMP-induced phosphorylation of SMAD1 in mouse embryonic fibroblasts

**Figure 3.**
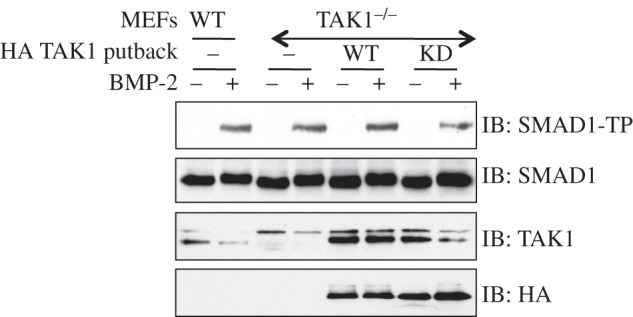
TAK1 does not affect BMP signalling in MEFs: wild-type (WT) or TAK1-deficient (TAK1^−/−^) MEFs stably reintroduced with a control vector (−) or vectors encoding HA-tagged TAK1 (WT) or a catalytically inactive TAK1 (D175A) mutant (KD) were treated with/without BMP-2 (25 ng ml^−1^, 60 min) prior to lysis. Extracts (20 μg) were resolved by SDS–PAGE and immunoblotted with phospho-SMAD1 (SMAD1-TP), total SMAD1 and TAK1 antibodies.

It has been reported that TAK1 impacts the BMP pathway in chondrocytes in part by directly phosphorylating the BMP-activated SMADs at their activating SXS motif [31]. Treatment of both WT MEFs and TAK1-deficient MEFs with BMP-2 led to phosphorylation of SMAD1 at Ser463 and Ser465 to the same extent (figure 3). Furthermore, restoration of WT TAK1 or KD TAK1 in TAK1-deficient MEFs did not alter the levels of BMP-induced phosphorylation of SMAD1, indicating that TAK1 does not mediate the BMP-induced phosphorylation of SMAD1 in MEFs (figure 3). It is therefore likely that any effect that TAK1 has on BMP signalling does not involve direct phosphorylation of SMAD proteins.

### 3.4. *RNAi* knockdown of MAP3K4 and MAP3K10 significantly suppress the TGFβ-induced phosphorylation and activation of p38 MAPK

**Figure 4.**
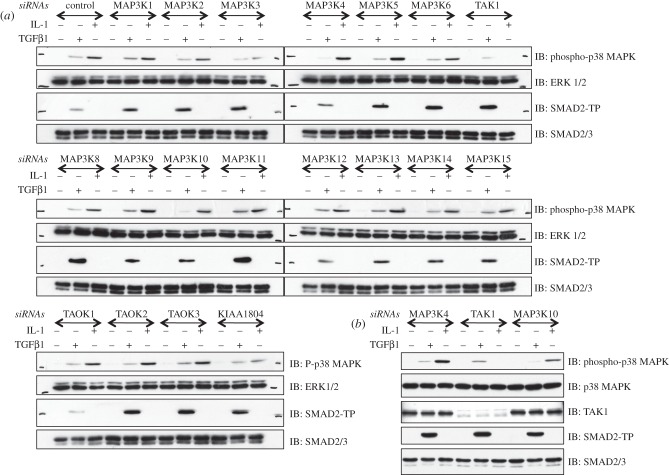
RNAi depletion of MAP3K4 and MAP3K10, but not TAK1, results in depletion of TGFβ-induced phosphorylation of p38 MAP3K: (*a*) human keratinocyte, HaCaT, cells were transfected with pools of 4 different siRNAs (75 pM of each siRNA per 10-cm diameter dish) targeted against the indicated mitogen-activated kinase kinase kinases (MAP3Ks). 48 h post-transfection, cells were left untreated or treated with TGFβ (50 pM; 45 min) or human IL-1β (5 ng ml^−1^; 10 min) prior to lysis. Extracts (10 μg) were resolved by SDS–PAGE and immunoblotted with antibodies against phospho-SMAD2 (SMAD2-TP), total SMAD2/3, phospho-p38 MAPK and total p38 MAPK. (*b*) As in (*a*) except that the indicated samples were run on the same gel for immunoblot analysis.

The surprising observations that TAK1 was not activated by TGFβ and did not mediate the TGFβ-induced p38 MAPK phosphorylation in MEFs and HaCaT keratinocytes suggested a role for other MAP3Ks in mediating the TGFβ-induced phosphorylation and activation of p38 MAPK. In order to address this in an unbiased manner, I undertook a comprehensive *RNAi*-based screening targeting the depletion of all of the human MAP3Ks individually to assess whether the depletion of specific MAP3Ks resulted in the inhibition of TGFβ-induced p38 MAPK phosphorylation. As control, I treated cells with human IL-1β, which is known to promote p38 MAPK phosphorylation through activation of TAK1 [32]. For each MAP3K target and a non-MAP3K control target, a pool of four *siRNAs* were transfected into HaCaT cells. As anticipated, the IL-1β-induced phosphorylation of p38 MAPK was substantially depleted only upon TAK1 (MAP3K7) knockdown, but was unaffected by knockdown of other MAP3Ks (figure 4*a*,*b*). The si*RNA* pool targeting TAK1 resulted in a robust depletion in expression of endogenous TAK1 protein (figure 4*b*). In all cases, the treatment of cells with TGFβ resulted in the phosphorylation of SMAD2 (figure 4*a*). The TGFβ-induced p38 MAPK phosphorylation was not affected by depletion of the majority of MAP3Ks, including TAK1 (figure 4*a,b*). However, the siRNA depletion of MAP3K4 (MEKK4) and MAP3K10 (MLK2) resulted in a significant reduction in the levels of TGFβ-induced phosphorylation of p38 MAPK (figure 4*a*), an observation that is further evident when MAP3K4, TAK1 and MAP3K10 *siRNA* screens are compared together (figure 4*b*). No immunoblotting antibodies were available to detect endogenous levels of MAP3K4 and MAP3K10 proteins (figure 4*b*).

### 3.5. *RNAi* knockdown of MAP3K10 in cells expressing catalytically inactive MAP3K4 (MAP3K4-KD) completely abolishes TGFβ-induced phosphorylation of p38 MAPK

**Figure 5.**
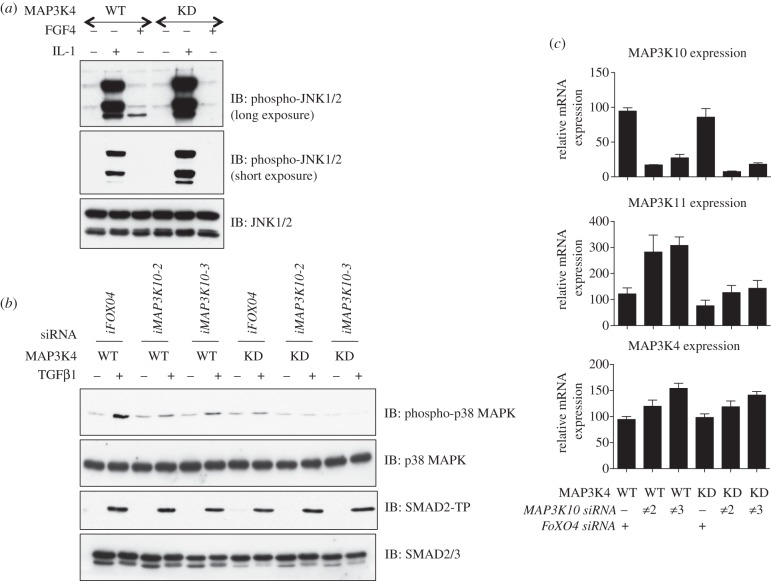
MAP3K4 and MAP3K10 drive the TGFβ-induced phosphorylation of p38 MAPK in MEFs. (*a*) MAP3K4^+/+^ (WT) MEFs or MEFs with a homozygous knockin of catalytically inactive mutant MEKK4^K1361R/K1361R^ (KD) were treated with or without mouse IL-1β (5 ng ml^−1^; 10 min) or FGF4 (10 ng ml^−1^; 60 min). Extracts (20 μg) were resolved by SDS–PAGE and immunoblotted with phospho-JNK and total JNK antibodies. (*b*) WT or KD MEFs were transfected with the indicated siRNA oligonucleotides. 48 h post-transfection, cells were treated with or without TGFβ (50 pM; 45 min) prior to lysis. Extracts (10 μg) were resolved by SDS–PAGE and immunoblotted with antibodies against phospho-p38 MAPK, total p38 MAPK, phospho-SMAD2 (SMAD2-TP) and total SMAD2/3. (*c*) As in (*b*) except that the cells transfected with the indicated *siRNAs* were left for 48 h and RNA isolated. Expression of MAP3K4, MAP3K10 and MAP3K11 mRNA was determined by RT-PCR analysis.

MAP3K4 (MEKK4) has previously been implicated in mediating SMAD-dependent activation of p38 MAPK [15]. This has, however, never been confirmed in cells derived from mice in which a catalytically inactive MAP3K4 (MAP3K4-KD) has replaced the WT protein [33]. I obtained WT and MAP3K4-KD MEFs, and investigated the TGFβ-induced phosphorylation of p38 MAPK in these MEFs. As reported previously, the FGF4-induced phosphorylation of one of the JNK isoforms was inhibited in MAP3K4-KD MEFs compared with the WT cells (figure 5*a*). Interestingly, IL-1α-induced phosphorylation of JNK1/2 was upregulated in MAP3K4-KD MEFs compared with the WT cells (figure 5*a*). The TGFβ-induced phosphorylation of p38 MAPK in MAP3K4-KD MEFs was inhibited by about 50 per cent compared with the WT MEFs (figure 5*b*; compare lanes 2 and 8), whereas the phosphorylation of SMAD2 was unaffected (figure 5*b*). These observations are consistent with the siRNA knockdowns of MAP3K4 (figure 4*a,b*) and indicate that while MAP3K4 has a role in mediating part of the TGFβ-induced phosphorylation of p38 MAPK, there is a role for further MAP3Ks in mediating the remainder of the TGFβ-induced p38 MAPK phosphorylation. Next, both WT and MAP3K4-KD MEFs were transfected with a control siRNA targeting *FOXO4* [34] or two independent *siRNAs* targeting MAP3K10, which resulted in the depletion of the MAP3K10 mRNA by more than 70 per cent (figure 5*c*). The depletion of MAP3K10 expression with two independent siRNAs in WT MEFs resulted in the inhibition of the TGFβ-induced p38 MAPK phosphorylation by about 50 per cent compared with the control (figure 5*b*), indicating that MAP3K10 also plays a role in mediating the TGFβ-induced phosphorylation of p38 MAPK. Interestingly, the depletion of MAP3K10 in MAP3K4-KD MEFs resulted in a complete loss of the TGFβ-induced p38 MAPK phosphorylation (figure 5*b*), suggesting that MAP3K4 and MAP3K10 are sufficient for the TGFβ-dependent phosphorylation of p38 MAPK. The depletion of MAP3K10 in either cell line did not alter the levels of TGFβ-induced phospho-SMAD2 (figure 5*b*). Interestingly, both siRNAs that yielded the depletion of MAP3K10 mRNA in both WT and MAP3K4-KD cells resulted in a significant increase in the expression of MAP3K11 transcripts (figure 5*c*).

### 3.6. TGFβ-induced p38 MAPK activation impacts CREB phosphorylation and transcription

**Figure 6.**
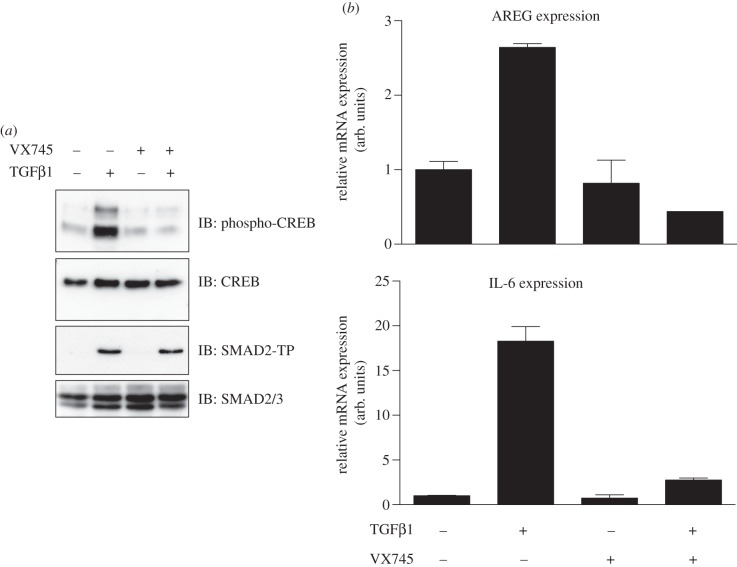
TGFβ-induced p38 MAPK activation impacts CREB phosphorylation and transcription. (*a*) Extracts (20 μg protein) from wild-type MEFs pre-incubated with 1 μM VX745 or DMSO for 1 h prior to stimulation with or without TGFβ (50 pM; 45 min) were resolved by SDS–PAGE and immunoblotted with the indicated antibodies. (*b*) As in (*a*) except that the cells were treated with or without TGFβ for 3 h prior to RNA isolation. Relative expression levels of AREG and IL-6 mRNAs were analysed by qRT-PCR. Data are represented as mean and error bars indicate standard deviation (*n* = 3).

I investigated whether TGFβ-induced activation of p38 MAPK impacted downstream signalling in MEFs. cAMP response element-binding protein (CREB) is phosphorylated at Ser133 upon activation of p38 MAPK and drives the expression of multiple target genes, including AREG and IL-6 [35–38]. In MEFs, TGFβ induced a robust CREB phosphorylation (figure 6*a*). This phosphorylation was inhibited by VX745, a selective and potent inhibitor of p38 MAPK (figure 6*a*) [39]. VX745 had no effect on TGFβ-induced phosphorylation of SMAD2 (figure 6*a*). By RT-PCR, I demonstrate that TGFβ induces the expression of AREG and IL-6 transcripts in MEFs (figure 6*b*). VX745 completely abolished the TGFβ-induced expression of AREG and IL-6 transcripts, suggesting that TGFβ-induced activation of p38 MAPK is necessary and sufficient for expression of these transcripts (figure 6*b*).

## 4. Discussion

In this study, I have demonstrated that TAK1 (MAP3K7) does not mediate the TGFβ-induced phosphorylation of p38 MAPK. By using two sets of MEFs from independently generated WT and TAK1-deficient mice, as well as TAK1-deficient MEFs restored with WT and catalytically inactive TAK1, I have demonstrated that the TGFβ-induced phosphorylation of SMAD2 and p38 MAPK remain unaffected in these cells. Furthermore, while the treatment of cells with IL-1α resulted in a robust activation of TAK1 in cells expressing WT TAK1, TGFβ stimulation did not enhance TAK1 activity. These findings contradict many reports implying a role for TAK1 in mediating the TGFβ-induced phosphorylation of p38 MAPK [10,11,17,25]. The report describing the discovery of TAK1 assigned its role in the TGFβ pathway based primarily on the ability of overexpressed TAK1 mutants to drive TGFβ-induced transcriptional reporter activity [17]. However, at the time many of the MAP3Ks were yet to be discovered. Even the discovery of SMAD proteins as key mediators of the TGFβ pathway was made a year after the first reported role for TAK1 in the TGFβ pathway [40,41]. A robust validation of the original findings with improved tools, including TAK1-knockout cells, was therfore long overdue. More recent reports addressing the possible role of TAK1 in TGFβ-induced phosphorylation of p38 MAPK have focused mainly on TRAF6 as an upstream activator of TAK1 [11,24]. While both reports describe how TRAF6 is essential for TGFβ-induced phosphorylation of p38 MAPK in MEFs and autoubiquitylation of TRAF6 in response to TGFβ recruits TAK1, neither report assesses the TGFβ-induced activation of TAK1 [11,24]. Furthermore, TRAF6 autoubiquitylation has been shown to be dispensable for IL1 and TLR-mediated activation of TAK1 [25]. It is therefore likely that TRAF6 could mediate the TGFβ-induced phosphorylation of p38 MAPK through another MAP3K.

By using an unbiased si*RNA* screen targeting all the human MAP3Ks, I have demonstrated that depletion of MAP3K4 (MEKK4) and MAP3K10 (MLK2) results in a significant inhibition of the TGFβ-induced phosphorylation of p38 MAPK, whereas the depletion of other MAP3Ks, including TAK1, did not significantly affect the p38 MAPK phosphorylation in response to TGFβ. As a proof that the *siRNA* screen used was an acceptable approach, I show that IL-1β-induced phosphorylation of p38 MAPK, which requires TAK1, is inhibited upon depletion of TAK1 by *siRNA* pool. However, some off-target effects of the *siRNAs* in the screen cannot be ruled out. Furthermore, because I was unable to verify the expression of intended targets by immunoblotting or RT-PCR, it is possible that certain targets may not have been depleted by certain *siRNA*s. Despite the limitations of the siRNA screen, it was very interesting that depletion of MAP3K4 resulted in the inhibition of TGFβ-induced p38 MAPK phosphorylation. MAP3K4 has previously been reported to mediate SMAD-dependent activation of p38 MAPK, in part by associating with an activator GADD45β, which is transcribed in response to TGFβ [15]. By using MEFs isolated from knockin mice expressing catalytically inactive MAP3K4, I have demonstrated for the first time that these MEFs display attenuated levels of p38 MAPK phosphorylation in response to TGFβ compared with the WT MEFs. This suggested that MAP3K4 was not sufficient to mediate the TGFβ-induced phosphorylation of p38 MAPK and implied a role for additional MAP3Ks in this process. When I depleted MAP3K10 with two independent *siRNAs* from MAP3K4-KD MEFs, the TGFβ-induced p38 MAPK phosphorylation was completely inhibited. However, depletion of MAP3K10 from WT MEFs, which still have intact MAP3K4, resulted in partial inhibition of the TGFβ-induced p38 MAPK phosphorylation. Taken together, it is clear that MAP3K4 and MAP3K10 are the two MAP3Ks that sufficiently mediate the TGFβ-induced phosphorylation of p38 MAPK in MEFs and HaCaT cells.

One of the most interesting observations from this study is that MAP3K10 (MLK2) lies upstream of p38 MAPK phosphorylation in response to TGFβ. MLK2 is a member of the mixed-lineage subfamily of kinases that includes three other members, namely MLK1 (MAP3K9), MLK3 (MAP3K11) and MLK4 (KIAA1804). It is interesting, however, that depletion of MLK1, MLK3 or MLK4 by siRNAs did not result in the inhibition of the TGFβ-induced p38 MAPK phosphorylation, indicating that MLK2 is sufficient for TGFβ-induced phosphorylation of p38 MAPK. In fact, depletion of MLK2 by two independent siRNAs, despite increasing the levels of MLK3 mRNA, inhibited TGFβ-induced p38 MAPK phosphorylation. It has been reported that overexpressed MAP3K11 (MLK3) was activated by TGFβ and was able to mediate the TGFβ-induced phosphorylation of p38 MAPK [12]. However, in my hands, the depletion of MAP3K11 in HaCaT cells did not result in any inhibition of the TGFβ-induced p38 MAPK phosphorylation. Furthermore, the depletion of MAP3K10 (MLK2) enhanced the levels of MAP3K11 transcripts in MEFs, but still resulted in the inhibition of TGFβ-induced p38 MAPK phosphorylation. Collectively, these results imply that MAP3K10 (MLK2), but not MAP3K11, mediates the TGFβ-induced phosphorylation of p38 MAPK. Investigation of TGFβ-induced p38 MAPK phosphorylation in MAP3K10-null MEFs would provide a definitive answer to the extent of its involvement in the TGFβ pathway. It will also be interesting to establish whether TGFβ activates the MLK family of protein kinases.

Because p38 MAPK lies at the heart of multiple pro-inflammatory signalling inputs, it has been an attractive target for inhibition to treat inflammatory diseases such as rheumatoid arthritis, psoriasis and chronic obstructive pulmonary disease [18,42]. As a result, numerous small molecule inhibitors of p38 MAPK, including VX745, have been developed and have entered clinical trials. Given the critical roles of p38 MAPK in mediating TGFβ-induced CREB phosphorylation and transcription (figure 6), as well as EMT [16] and cell death [13,43], the use of these inhibitors in a clinical context may have consequences on the TGFβ responses as well. Depending on different biological contexts, p38 MAPK inhibitors may prove to be useful as inhibitors of TGFβ-induced metastasis (by inhibiting EMT) or may prove less useful by promoting tumour proliferation through blocking TGFβ-induced apoptosis.

## 5. Material and methods

### 5.1. Materials

Antibodies recognizing total p38 MAPK, phospho-p38 MAPK (Thr180 and Tyr182), total SAPK/JNK, phospho-SMAD1 (Ser463/465), phospho-SMAD2 (Ser465/467), total SMAD2/3, phospho-CREB (Ser133), total CREB and MAP3K3 (MEKK3) were purchased from Cell Signaling. Antibodies recognizing phospho-JNK1/2 (Thr183/Tyr185) and Lipofectamine 2000 reagent were purchased from Invitrogen. Antibodies recognizing TAK1, MAP3K8, MAP3K4, MAP3K10 (N- and C-terminus) and MAP3K11 were purchased from Santa Cruz Biotechnology. ^32^P γ-ATP was from Perkin-Elmer. BMP-2, TGFβ1 and mouse IL-1α were from R&D Biosystems. Polybrene, Puromycin, DMSO and Tween-20 were from Sigma. Antibody recognizing SMAD1 was generated in sheep against a glutathione S-transferase-tagged SMAD1(144–268) fragment and affinity purified [44]. Immunoprecipitating anti-TAB1 antibody was generated in sheep against a His-TAB1 protein and affinity purified. Pre-immune IgG was purified and used as control. Human IL-1β was expressed in bacteria. These antibodies and proteins were generated at the Division of Signal Transduction Therapy at the University of Dundee (UK). TAK1-deficient (TAK1^−/−^) and corresponding WT MEFs were a generous gift from S. Akira (Osaka University, Japan) [32]. Independently generated TAK1-null and corresponding WT MEFs were obtained from Prof. Sankar Ghosh (Columbia University, New York, NY) [29]. The MAP3K4-KD MEFs, derived from mouse embryos in which the WT protein is replaced by the catalytically inactive MA3K4 (K1361R) mutant, and the corresponding WT MEFs, were a generous gift from G. Johnson (University of North Carolina, NC) [33]. VX745 was generated by Natalia Shpiro (University of Dundee).

### 5.2. cDNA constructs, siRNA sequences and RT-PCR primers

N-terminal HA-tagged WT TAK1 (TAK1-WT) or catalytically inactive mutant of TAK1 (TAK1(D175A); TAK1-KD) was cloned into a pBABE-puro retroviral vector (Cell Biolabs). pCMV-Gag-Pol and pCMV-VSVG constructs were purchased from Cell Biolabs. All DNA constructs used were verified by DNA sequencing, performed by DNA Sequencing and Services (MRCPPU, College of Life Sciences, University of Dundee; www.dnaseq.co.uk) using Applied Biosystems Big-Dye v. 3.1 chemistry on an Applied Biosystems model 3730 automated capillary DNA sequencer. In order to knockdown the indicated panels of human MAP3Ks (figure 4), a pool of four siRNA duplexes designed against each target, and a pool of four control scramble siRNA oligonucleotides, were purchased from Dharmacon (sold as SMARTpool siRNA). The target sequence for each individual siRNA in the pool was not provided. The sequences for sense strands for mouse MAP3K10 siRNAs (purchased from Sigma) are as follows: siRNA1 (5′–3′): CGGUUGAGGGCCAUUCGAU(dT)(dT) and siRNA3 (5′–3′): CUGACACGGUGCUCAAGAU(dT)(dT).

RT-PCR primers used in the study are as follows (all 5′–3′): mouse MAP3K4-F: CTAAGTCCTATGATAACGTCATGC; R: TGAAATCGAATCTCCTTCATGG; mouse MAP3K10-F: CTGGTGATGGAATATATGCTCG; R: CCAGGATTAGGATGTTGATGG; mouse MAP3K11-F: CCCTTCAACTCTGAATCTAATCC; R: CGAAGTGGATCTACTTGAAGC; mouse GAPDH-F: TATGATGACATCAAGAAGGTGG; R: CATTGTCATACCAGGAAATGAG; mouse IL-6-F: TTCCATCCAGTTGCCTTCTTG; R: AGGTCTGTTGGGAGTGCTATC; mouse AREG-F: CGACAAGAAAACGGGACTG; R: AACTGGGCATCTGGAACC; mouse 18S-F: GTAACCCGTTGAACCCCATT; R: CCATCCAATCGGTAGTAGCG.

For real-time PCR, isolated RNA (1 µg) was used to prepare cDNA using I-script kit (BioRad). Reactions from three biological replicates were performed in triplicates of 20 µl each, including 0.5 per cent cDNA, 1 µM forward and reverse primers, and 50 per cent SYBR Green (Quanta). RT-PCR was performed using a standard protocol in a CFX 384 real-time system (BioRad). Data were normalized to a housekeeping gene (GAPDH or 18S). The data were analysed as reported previously [7,45].

### 5.3. Generation of TAK1-deficient mouse embryonic fibroblasts stably expressing TAK1-WT or TAK1-KD

Retroviral pBABE-puro constructs (1 µg each) encoding an HA tag or an N-terminally HA-tagged TAK1-WT or TAK1-KD were co-expressed with CMV-Gag/Pol (0.9 µg) and CMV-VSVG (0.1 µg) constructs in HEK-293T cells. Retroviruses were collected 48 h-post transfection from the culture media by filtering it through 0.45 µm filters onto sterile falcon tubes. TAK1-deficient MEFs, plated at approximately 40 per cent confluent, were infected by transferring the filtered retroviruses directly onto the cells, and 5 µg ml^−1^ Polybrene reagent was added to aid infection. 24 h post-infection, cells were cultured in the presence of 2 µg ml^−1^ puromycin for selection of infected cells. Western blots with TAK1 or HA antibodies were used to confirm successful infection of appropriate targets. The retroviral means of generating stable cell lines ensures expression of target proteins at levels that are comparable with the endogenous levels of expression in similar cells that express the proteins naturally.

### 5.4. Cell culture, stimulation and lysis

HaCaT cells, two independent sets of WT MEFs and TAK1-deficient MEFs, MAP3K4^+/+^, and MAP3K4-KD MEFs were cultured in dishes of 10 cm diameter in Dulbecco's modified Eagle's medium (DMEM) supplemented with 10 per cent foetal bovine serum (FBS), 1 per cent penicillin/streptomycin mix and 2 mM l-glutamine (D10F). All cells were grown under a humidified atmosphere with 5 per cent CO_2_ at a constant temperature of 37°C. TAK1-deficient MEFs stably expressing a control vector or WT TAK1 (TAK1-WT MEFs) or KD TAK1 (TAK1-KD MEFs) were cultured as above except that the medium was supplemented with 2 µg ml^−1^ puromycin. Individual or pools of siRNAs (300 pmoles final per 10 cm 50% confluent dish) were transfected using Lipofectamine 2000 reagent as described previously [34,46]. Cells were cultured in DMEM containing 0.1 per cent FBS for 16 h prior to treatment with appropriate ligands. Unless stated otherwise, cells were treated with TGFβ (50 pM final) or BMP-2 (25 ng ml^−1^ final) for 45 min, and human IL-1β (1 µg ml^−1^ final) or mouse IL-1α (5 ng ml^−1^ final) for 10 min. Cells were then washed once with ice-cold PBS and lysed in 0.5 ml ice-cold complete lysis buffer (50 mM Tris–HCl pH 7.5, 1 mM EGTA, 1 mM EDTA, 1% Triton X-100, 1 mM sodium orthovanadate, 50 mM sodium flouride, 5 mM sodium pyrophosphate, 0.27 M sucrose, 5 mM β-glycerophosphate, 0.1% (v/v) 2-mercaptoethanol, 0.5 µM microcystin-LR, one tablet per 25 ml of complete protease inhibitor cocktail). The extracts were cleared by centrifuging at 16 000*g* at 4°C for 10 min and snap-frozen in liquid nitrogen for storage at −80°C or processed immediately. For RNA isolation, cells were processed using Nucleospin II RNA Isolation kit (Macherey-Nagel) according to the manufacturer's instructions.

### 5.5. SDS–PAGE and immunoblotting

Cleared cell extracts (20 μg) were heated at 95°C for 5 min in 1× SDS sample buffer (62.5 mM Tris–HCl pH 6.8, 10% (v/v) glycerol, 2% (w/v) SDS, 0.02% (w/v) bromophenol blue and 1% (v/v) β-mercaptoethanol), resolved on a 10 per cent polyacrylamide gel by electrophoresis and transferred to nitrocellulose membranes. Membranes were blocked in TBS-T buffer (50 mM Tris–HCl pH 7.5, 0.15 M NaCl and 0.1% (w/v) Tween-20) containing 10 per cent (w/v) non-fat milk for 1 h at room temperature. The membranes were then incubated with the indicated antibodies (diluted in TBS-T containing 10% (w/v) milk) for 16 h at 4°C, washed 2 × 10 min in TBS-T buffer, probed with the HRP-conjugated secondary antibodies (diluted 1 : 5000 in TBS-T/5% milk) for 1 h at room temperature, and washed 3 × 10 min in TBS-T buffer. Enhanced chemiluminescence reagent was used to detect the signals.

### 5.6. *In vitro* TAK1 kinase assay

TAK1 assays were performed as described previously [30]. Briefly, TAK1 associated with TAB1 was immunoprecipitated from cell extracts (1 mg total protein) using 2 µg of anti-TAB1 antibody coupled to 5 µl of protein G-sepharose beads for 2 h at 4°C. The immunoprecipitates were washed twice with 1 ml of lysis buffer containing 0.5 M NaCl followed by two further washes with 1 ml of 50 mM Tris–HCl pH 7.5, 0.1 mM EGTA and 0.1% (v/v) 2-mercaptoethanol. The TAK1 activity in the immunoprecipitates was assessed by its ability to activate MKK6 as judged by its activation of p38α MAPK. The activity of p38α MAPK was then assayed by measuring its ability to phosphorylate MBP as described previously [30].
